# A new esthetic fiber-reinforced polymer composite resin archwire: a comparative atomic force microscope (AFM) and field-emission scanning electron microscope (FESEM) study

**DOI:** 10.1186/s40510-014-0039-8

**Published:** 2014-05-30

**Authors:** Chai Kiat Chng, Kelvin Foong, Narayan H Gandedkar, Yiong Huak Chan, Chong-Lin Chew

**Affiliations:** Department of Craniofacial Dentistry, Division of Plastic, Reconstructive, and Aesthetic Surgery, KK Women’s and Children’s Hospital, Singapore, 229899 Singapore; Department of Orthodontics, Faculty of Dentistry, National University of Singapore, Singapore, 119077 Singapore; Yong Loo Lin School of Medicine, National University Health System, Singapore, 119228 Singapore; Graduate School of Dental Studies; Centre for Advance Dental Education, Faculty of Dentistry, National University of Singapore, Singapore, 119077 Singapore

## Abstract

**Background:**

Fiber-reinforced polymer composite (FRPC) archwires could provide an esthetic solution to conventional orthodontic archwires. This study was carried out with the following aims: (1) to compare the sliding friction of FRPC archwire with nickel titanium archwire using various archwire-bracket combinations and (2) to determine the correlation between surface roughness and friction of the FRPC and NiTi archwires.

**Methods:**

Four different brackets (Gemini® (Gemini-3M Unitek, St. Paul, MN, USA), ICE® (ICE-Ormco- Orange, CA, USA), Clarity® (Clarity-3M Unitek, St. Paul, MN, USA), and SmartClip® (SmartClip-3M Unitek, St. Paul, MN, USA)) in combination with FRPC wires and NiTi wires (0.018 in) were studied for archwire friction with simulated wear and surface roughness using scanning electron microscope (SEM) and atomic force microscope (AFM), respectively. Statistical analysis of frictional wear generated and surface roughness between the various archwire and bracket groups was evaluated by one-way ANOVA at 5% level. Least significant difference (LSD) multiple comparisons were used to determine the archwire-bracket group difference.

**Results:**

Gemini®-FRPC group generated the highest frictional wear (mean, 313.10; SD, 802.59) and ICE®-FRPC group produced the highest roughness values among all the groups tested (Ra = 496.13 nm, RMS = 635.49 nm). No correlation was found between frictional wear and surface roughness of the archwires of the various groups.

**Conclusions:**

FRPC archwire shows promise in its application as an esthetic aligning archwire. However, further research and refinement in its manufacture would be necessary to fully realize its potential as an esthetic archwire.

**Electronic supplementary material:**

The online version of this article (doi:10.1186/s40510-014-0039-8) contains supplementary material, which is available to authorized users.

## Background

With the advent of increasing number of adults opting for orthodontic treatment, the development of orthodontic appliances with ample emphasis on esthetics coupled with optimal performance has become an exceedingly essential goal or rather necessity of the day. Through composite technology, an esthetic wire has been developed from continuous fibers (E-glass fiber) and epoxy polymer matrix (tube shrinkage technique), giving rise to a prototype, fiber-reinforced polymer composite (FRPC) archwire (BioMers Products LLC, Naples, FL, USA), which is potentially suitable for use in orthodontics [1]. Literature suggests that the bending stiffness and strength of the FRPC archwire were comparable to those archwires routinely used in orthodontic treatment [2–4].

Springback, stiffness, formability, resilience modulus, biocompatibility, and low friction are some of the desirable characteristics of an archwire for optimum mechanical performance during orthodontic treatment [5, 6]. FRPC archwire's tensile and three-point bending tests have shown that the archwire's mechanical properties are comparable to nickel titanium [1].

In the past, coated esthetic wires have been used as archwires, fixed retainers [7], and to reinforce anchorage [8], but such wires have higher friction (archwires), and the esthetic coating tend to dehisce over a period of time [9–17]. Also, coated wires, being opaque, cause no tooth color transmission through these wires. The FRPC archwire, being translucent in nature, allows tooth color transmission, thereby improving esthetics. Aforementioned phenomenon is particularly beneficial in cases where ceramic/tooth colored brackets are used in conjunction with FRPC wires for orthodontic treatment. Moreover, allergic reactions to nickel [18–21], a metallic ion found commonly in contemporary metallic archwires, is averted with the FRPC archwire.

Although, FRPC can offset the unpleasant appearance of the metallic archwires, the frictional characteristics of the wire cannot be overlooked, as friction plays a critical role throughout the course of orthodontic treatment [22, 23]. At various stages of orthodontic treatment, the mechanical properties required by the archwire differ; different fiber material, fiber content, and fiber arrangement can modify the mechanical properties of the wire to suit the mechanical requirements of the archwire at various stages of treatment.

Moreover, low friction is one of the optimal desirable properties of an ideal orthodontic archwire [24, 25]. The success or failure of fixed appliance orthodontic treatment is greatly influenced by the frictional properties of the materials used and how friction is applied and controlled, in the due course of treatment.

In this study, the friction and wear characteristics of a novel FRPC archwire were evaluated against nickel titanium (NiTi) archwire with various commercially available bracket systems with the following aims and objectives:

1. to determine sliding friction of FRPC archwire with nickel titanium archwire using various archwire-bracket combinations
2. to determine the correlation between surface roughness and friction of the FRPC and NiTi archwires.

Therefore, the null hypothesis tested was that there would be no difference in sliding friction and surface roughness of FRPC archwire and nickel titanium archwires when combined with different bracket systems.

## Methods

The 0.018-in. FRPC archwire (Translucent Archwire, BioMers Products LLC) was compared against 0.018-in. NiTi archwire (Super Elastic NiTi, International Orthodontic Services Inc, Houston, TX, USA) (Table 1). The two archwires were tested against four different commercially available brackets having 0.022 × 0.028-in. slot size (Table 2). For the purpose of standardization, only the upper right first premolar brackets having similar torque and angulation with −7° torque and 0° angulation, respectively, were used.

**Table 1:** **Brands, manufacturing companies, configurations, and composition of various brackets used in this study**

Brand	Manufacturer	Configuration	Composition
Gemini	3M Unitek, St. Paul, MN, USA	True twin	Stainless steel
Clarity	3M Unitek, St Paul, MN, USA	True twin	Polycrystalline metal-reinforced ceramic
Inspire Ice	Ormco, Orange, CA, USA	True twin	Monocrystalline ceramic
SmartClip	3M Unitek, St Paul, MN, USA	True twin	Stainless steel
		Self-ligating	

True-twin bracket configuration was selected with ceramic and stainless steel composition. The selection of bracket material composition (stainless steel and ceramic (polycrystalline and monocrystalline)) was deliberate in order to encompass brackets with the commonest material composition in this study.

**Table 2:** **Orthodontic archwires used in the study**

Wire	Wire size (in.) and brand	Composition	Manufacturer
Translucent composite	0.018	Mixture of cured copolymers, Bis-EMA, TEGDMA, and glass fibers	BioMers Products LLC, Naples, FL, USA
Nickel titanium	0.018	52% Ni, 45% Ti, and 3% Co	International Orthodontic Services Inc., Houston, TX, USA

Wires of 0.018-in. dimension were studied for friction and roughness with various archwire-bracket combinations. Bis-EMA, ethoxylated bisphenol A glycol dimethacrylate; TEGDMA, triethylene glycol dimethacrylate.

### Friction test

**Figure 1:**
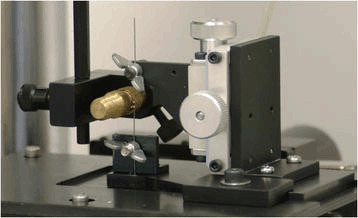
**Experimental setup of the universal testing machine with bracket and archwire mounted on a customized jig.** Customized jig allowed the bracket to traverse a specified distance over a specific period of time.

The frictional test of each archwire-bracket interface was carried out using a universal testing machine (Model 5848 Micro Tester, Instron Corporation, Norwood, MA, USA). To avoid contamination, all the brackets and wires were cleaned with alcohol swabs before use, and utmost care was exercised during the handling of the same. The test wires were supplied as straight in shape without preformed archwire shapes to avoid trifocal ellipse of the preformed archwires. Stainless steel ligature (PL 1010 Ligature Wire, GAC International, Commack, NY, USA) was twisted until the test wire was firmly secured in the bracket slot and then untwisted three turns to avoid archwire-bracket binding. This was carried out for all the test wire and bracket combinations except for self-ligating brackets. Furthermore, the apparatus was set using a customized jig (Figure 1), such that the bracket traversed 1 mm of test wire at the rate of 0.5 mm/min moving upwards and then downwards for 2 min with each cycle lasting for 4 min with a static load cell ±1 kN (100 kgf, 225 lbf) having 0.025% accuracy (series number S11900, Instron Corporation). A total of 10 cycles were carried out for each test wire and bracket combination, with each test lasting for 40 min. In order to rule out inertia caused by the change in crosshead direction during the last 10 s of each end, only the magnitude of the readings was recorded while discarding the readings obtained at the first and last 10 s of each 2-min cycle.

Five tests were carried out for each bracket and archwire combination. A total of eight archwire and bracket combinations were formed with two archwires and four different brackets. The values of the frictional wear of each archwire-bracket test were captured from the universal testing machine to dedicated software (Merlin Software, Instron Corporation); this software was used to calculate the frictional values. Field emission scanning electron microscope (FESEM) and atomic force microscope (AFM) studies were conducted for qualitative surface analysis and quantitative analysis of the archwires, respectively.

### FESEM study

The scanning electron microscope (SEM) study was carried out with a field-emission scanning electron microscope (FESEM) (Quanta 200F, FEI, Hillsboro, OR, USA). One test wire from each of the wire-bracket combination was used for the FESEM analysis. One untested FRPC wire and one untested NiTi wire from each group formed the control sample. Before subjecting the specimens for FESEM analysis, all the specimens were prepared by placing them individually into an auto fine coater (JEOL Model JFC 1600, Tokyo, Japan). The samples were gold coated for 60 s using a 10-mA current. The area for FESEM scanning was localized by determining the middle of the friction test site and the area 1 mm above and below this mid-point along which the length of the wire was scanned. This area was scanned and analyzed with magnification factor of ×220 to ×5,000.

### AFM study

AFM (Dimension™ 3100, Nanoscope IIIa, Digital Instruments Inc., Santa Barbara, CA, USA) with ‘RTESP Tap300’ Metrology Probe (Veeco, Plainview, NY, USA) was used to perform the surface analysis of the archwires in this study. The microscope raster scans were obtained by placing the probe over the sample while measuring the local properties (height, optical absorption, and magnetism). Tapping Mode™ (Nanocsope IIIa, Digital Instruments Inc.) was used to analyze the surface topography of the test wires. For the purpose of comparison, the root mean square (RMS) roughness was chosen to characterize the topography of the test wires after the test wires have undergone frictional testing with the universal testing machine. For AFM analysis, four test wire-bracket combinations were randomly selected from the original sample of five test wire and bracket combinations. The 1-mm test area created by universal testing machine was visually scanned and localized using the nanoscope, and three surface scans were taken from the area: one at the middle and two more at the ends of the test area. The RMS roughness of these surface areas was determined along with mean and standard deviation [22].

### Statistical analysis

All the statistical analyses were carried out using the statistical package for social sciences (SPSS version 14, SPSS Inc., Chicago, IL, USA) software. Based on the preliminary data and a test power analysis of 0.85, the sample size calculation showed that at least three specimens are sufficient to detect a 10% difference with a significance level of 5%. The mean and standard deviation of the frictional wear and surface roughness were evaluated. A one-way ANOVA was carried out to compare the friction wear and surface roughness between groups and also within groups. Multiple comparisons were performed using least significant difference (LSD) with a *post hoc* Bonferroni adjustment.

## Results

### Quantitative analysis of friction study

From descriptive statistics of frictional wear (Table 3), it can be seen that the frictional wear generated showed a range of values among the different archwire and bracket combinations. The highest frictional wear generated was with the Gemini-FRPC group (313.10 ± 802.59 N). The results also indicated that some archwire and bracket combinations showed less friction (SmartClip-NiTi, −6.94 ± 48.27 N) at the end of testing, indicating that the coefficient of friction was possibly reduced after frictional wear.

**Table 3:** **Descriptive statistics of eight bracket-archwire combinations tested for friction and roughness (AFM)**

Bracket-archwire combinations	Friction (N)	Roughness
	(mean ± SD)	(average RMS)
		(mean ± SD)
Clarity-FRPC	−27.88 ± 33.59	324.96 ± 143.76
Clarity-NiTi	−22.83 ± 34.74	166.88 ± 39.66
Gemini-FRPC	313.10 ± 802.59	310.45 ± 70.46
Gemini-NiTi	75.41 ± 159.06	183.95 ± 44.53
ICE-FRPC	−71.13 ± 17.08	398.47 ± 249.04
ICE-NITi	−28.48 ± 21.44	206.55 ± 70.74
SmartClip-FRPC	27.48 ± 72.85	321.16 ± 27.52
SmartClip-NiTi	−6.94 ± 48.27	172.80 ± 52.49

AFM, atomic force microscope; N, Newton; RMS, root mean square; FRPC, fiber-reinforced polymer composite; NiTi, nickel titanium.

There was no significant difference noted for the frictional wear generated between the various archwire and bracket groups (*P* = 0.542). Also, no statistical significance was noted within individual archwire-bracket groups (Table 3).

Multiple comparisons of the groups showed significant difference in frictional wear. LSD multiple comparison revealed statistical significance (*P* < 0.05) when comparing the Gemini-FRPC with the ICE-FRPC group. No other groups showed any significant difference.

### Qualitative analysis of SEM study

#### Control FRPC and NiTi wires

Both FRPC and NiTi control wires appeared to be relatively smooth with minimal surface defects. Inclusions noted on surfaces were probably not inherent of the surface structures, but could be a result of contamination during handling of these wires.

### Tested NiTi wires

**Figure 2:**
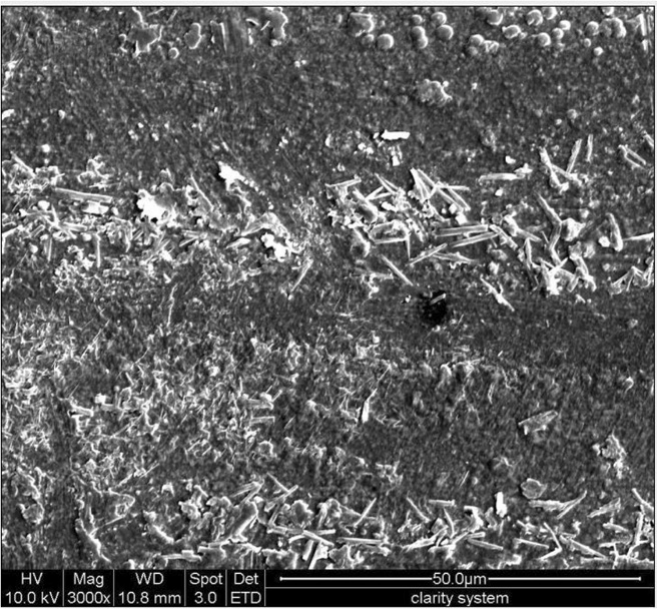
**FESEM image of a FRPC wire from the Clarity-FRPC group showing crystal-like deposits.** These crystal-like deposits were present in large amount in Clarity-FRPC group than in any other tested groups (original magnification ×3,000, bar = 50 μm).

On the tested NiTi wires, ‘scratch’ marks were evident not only on the horizontal pattern but also on the vertical one. Clarity-NiTi and Clarity-FRPC (Figure 2) groups showed distinct crystal-like deposits on their surfaces. These crystals appeared more on Clarity-FRPC specimens than on any other wire-bracket combinations tested.

### Tested FRPC wires

**Figure 3:**
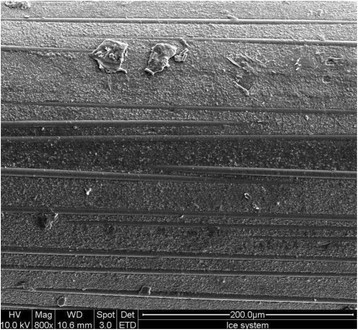
**FRPC tested wires with ICE bracket system.** The wire-bracket combination showed wear of surface polymer composite and exposure of underlying fibers. Original magnification ×800, bar = 200 μm).

**Figure 4:**
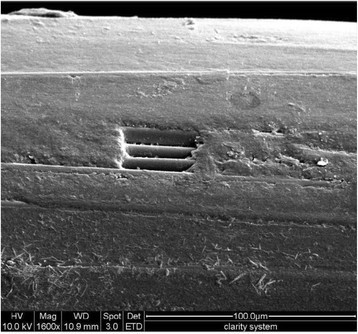
**FRPC tested wires of Clarity-FRPC group.** The wires showed bulk fracture of the polymer composite, giving rise to pit defect on the wire (original magnification ×1600, bar = 100 μm).

The surface of the polymer composite was worn away in many of the wires, which exposed the underlying fibers within the FRPC wire (Figure 3). In some areas of wires tested, the bulk fracture of the polymer composite was observed, giving rise to a pit defect on the wire (Figure 4).

### Quantitative analysis of surface roughness from AFM study

The surface roughness values were generally higher in bracket-archwire combinations with the FRPC archwire. The ICE-FRPC group produced the highest roughness values among all the groups tested. Untested wires were also analyzed for roughness, where untested FRPC showed average roughness of RMS = 321.89 nm and untested NiTi wires showed roughness of RMS = 261.5689 nm.

**Figure 5:**
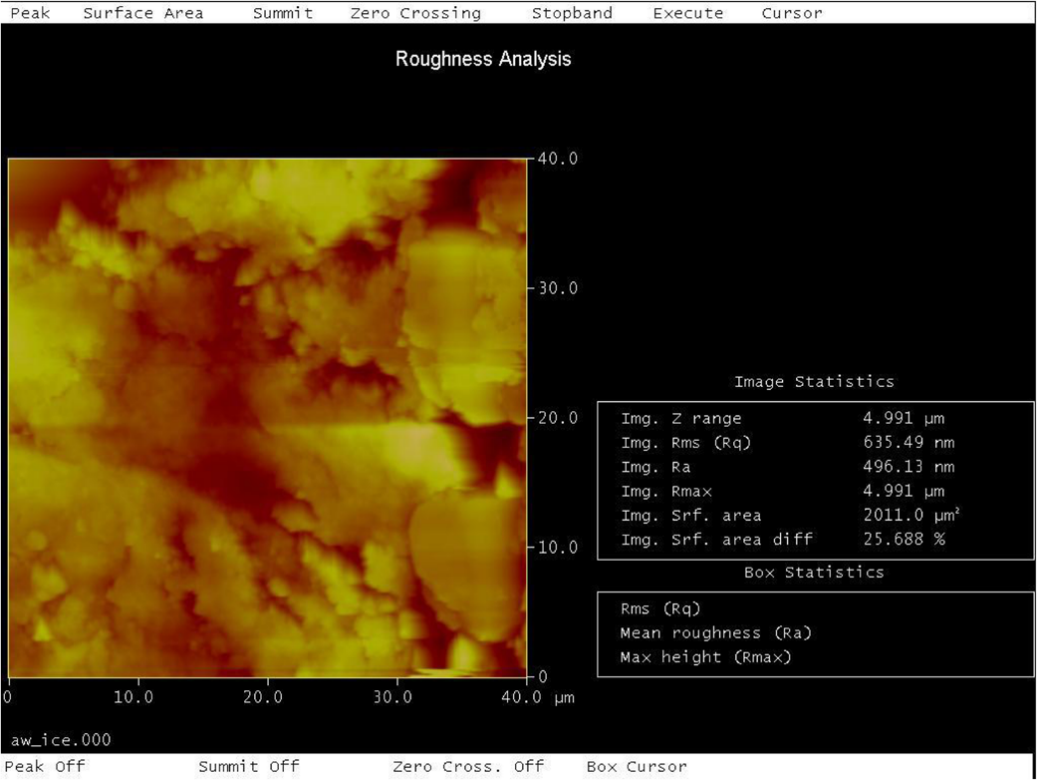
**Image showing surface roughness analysis of FRPC wire of ICE-FRPC group**. The FRPC wire showed maximum roughness with Ra = 496.13 nm, RMS = 635.49 nm, and surface area difference of 25.6% (original magnification ×40, 40 μm).

**Figure 6:**
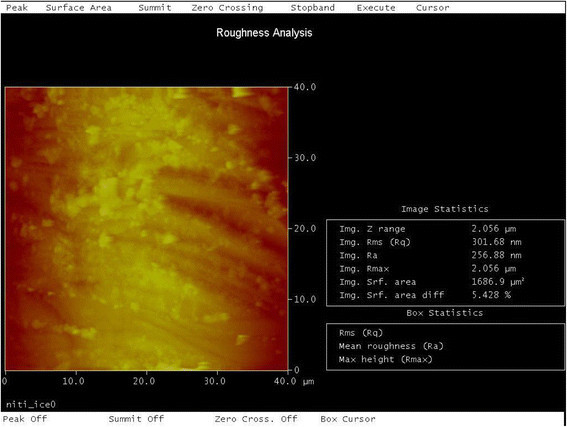
**Image showing surface roughness analysis of NiTi wire of NiTi-ICE group.** NiTi wire showed roughness with Ra = 256.88 nm, RMS = 301.68 nm, and surface area difference of 5.42% (original magnification ×40 40 μm).

**Figure 7:**
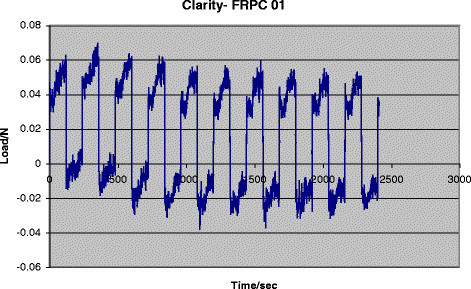
**Studied frictional load (N) vs time (s) graph of one of the tested group.**

There was significant difference between the different groups of the surface roughness of the archwires (*P* = 0.044). No intra-group difference was noted. In multiple group comparison LSD, the ICE-FRPC group (Figure 5) showed a statistically significant roughness (*P* < 0.05) when compared to ICE-NiTi (Figure 6), Clarity-NiTi, Gemini-NiTi, and SmartClip-NiTi groups. A graph of the studied frictional load (N) vs time (s) of one of the tested groups is shown in Figure 7.

## Discussion

The null hypothesis of the study was rejected as different archwire-bracket combinations produced different degrees of surface roughness, with the ICE-FRPC group showing the highest roughness value among all the groups tested (Table 3). The FRPC wire tested in this study has been designed and manufactured with the intention to function as wire used in the initial alignment phase of orthodontic treatment with high esthetic value. The use of equivalent-diameter contemporary nickel titanium archwires for comparison of friction and surface roughness is therefore appropriate. Four different brackets and two archwire materials were combined to give eight different bracket-archwire interface combinations to quantify the amount of sliding friction and surface roughness with SEM and AFM, respectively. The wear patterns created on the surface of each archwire material with the eight different bracket-archwire interfaces were determined and were co-related with the sliding friction and surface roughness.

### Sliding friction

Frictional wear from the friction studies showed no significant difference between the groups. From the LSD multiple comparison, only the ICE-FRPC group showed statistical significance when compared to the Gemini-FRPC group. However, a *post hoc* Bonferroni adjustment for multiple comparisons showed that the Gemini-FRPC group showed no statistical significance. All other FRPC combination groups did not show any statistical significance in the frictional testing. This would indicate that the FRPC wire's performance was similar to that of the NiTi wire during friction testing.

### Scanning electron microscopy of surface wear

Clarity bracket (ceramic) and wire combination showed crystal-like structural deposits on the tested wires. However, the same was not noted in ICE (ceramic) bracket-wire combination. Though both (Clarity and ICE) are ceramic brackets, the structural deposits were not noted in the ICE bracket-wire combination; this could be attributed to the structural differences in both brackets. Clarity is polycrystalline and ICE is monocrystalline; polycrystalline ceramic brackets usually have structural imperfections, and the incorporation of impurities as a result of manufacturing process makes it structurally weaker than monocrystalline ceramic brackets [26]. Microfractures may also have occurred in the Clarity bracket, giving rise to fragments of deposits on the surface of the tested wires. All the tested bracket slots were reobserved for asperities arising as a result of wire abrasion and also observed for bracket slot color changes arising from the metallic wire. Neither asperities nor color changes were noted, confirming the fragments of deposits to be bracket microfractures.

NiTi wires showed horizontal wear tracks; these finding were congruent with the findings noted by Al-Khatib et al. [18]. Scratches, groves, and oxidized layer were noted which are believed to be caused by the wear mechanisms that are active in the sliding contacts between the bracket and archwire, inducing a competition between abrasive wear and oxidational wear processes. Along with the aforementioned findings, vertical track marks were also noticed which could be due to the result of ligation of the wire with stainless steel ligation wires.

FRPC wires also appeared to have fibers exposed after frictional testing. The exposure of the fibers did not significantly affect roughness or frictional values. Discrete penetration separated by sections of undamaged coating was observed in one of the wires. This was also noted by Zufall et al. [9]. The cause of the bulk fracture of composite is yet to be ascertained, but a likely reason would be an area of weakness due to premature polymerization of the composite during the manufacturing process or a void that was present just below the area of penetration.

### Atomic force microscopy of surface roughness

A commercial AFM (Dimension 3100, Nanocsope IIIa, Digital Instruments Inc.) was used to perform the surface analysis of the archwires. The AFM is one of the types of scanned-proximity probe microscopes (SPMs). The tip used in this study was the RTESP Tap300 Metrology Probe. SPMs work by measuring a local property, such as height, optical absorption, and magnetism, with a ‘probe’ or ‘tip’ placed very close to the sample. The small probe-sample separation (on the order of the instrument's resolution) makes it possible to take measurements over a small area. To acquire an image, the microscope raster scans the probe over the sample while measuring the local property in question. The resulting image resembles an image on a television screen in that both consist of many rows or lines of information placed one above the other.

Unlike traditional microscopes, scanned-probe systems do not use lenses; hence, the size of the probe rather than diffraction affects the resolution. The tip is very fine (<100 nm) and is mounted on a thin cantilever of approximately 100 μm in length. The cantilever has a deflection modulus between 0.001 and 100 N/m, bringing the tip into a distance of about 10 to 100 nm of the specimen surface. This interaction between specimen surface and tip results in forces that range between 10^−11^ and 10^−6^ N. Due to this interaction, the cantilever was bent, and the vertical deflection of its end was registered by a deflection sensor. The AFM thus operates by measuring attractive or repulsive forces between the tip and the sample. There are many modes of scanning that can be used by the AFM. The three most common are the (1) contact, (2) non-contact, and (3) Tapping Mode™. Tapping Mode™ AFM, the most commonly used of all AFM modes, is a technique that maps topography by lightly tapping the surface with an oscillating probe tip. The cantilever's oscillation amplitude changes with sample surface topography, and the topography image is obtained by monitoring these changes and closing the *z* feedback loop to minimize them. TappingMode™ has become an important AFM technique, as it overcomes some of the limitations of both contact and non-contact AFM. By eliminating lateral forces that can damage soft samples and also reduce image resolution, TappingMode™ allows routine imaging of samples once considered impossible to image with AFM, especially in contact mode. Another major advantage of Tapping Mode™ is related to the limitations that can arise due to the thin layer of liquid that forms on most sample surfaces in an ambient imaging environment, i.e., in air or some other gas. The amplitude of the cantilever oscillation in TappingMode™ is typically on the order of a few tens of nanometers, which ensures that the tip does not get stuck in this liquid layer. The amplitude used in non-contact AFM is much smaller, as different forces are being measured. As a result, the non-contact tip often gets stuck in the liquid layer unless the scan is performed at a very slow speed. In general, TappingMode™ is much more effective than non-contact AFM for imaging larger scan sizes that may include large variations in sample topography. TappingMode™ can be performed in gases, liquids, and some vacuum environments. For this part of the study using the AFM, the TappingMode™ was used to analyze surface topography of the test wires.

RMS roughness was chosen to characterize the topography of the test wires. Roughness is a generic term that is used to indicate unevenness, whereas RMS is a quadratic mean, a statistical measure of the magnitude of a varying quantity. It is especially useful when variants are positive and negative and are taken from a sample surface, but not from the entire surface. In our study, the peak and valley on the sample surface measured indicates the positive and negative variants. Hence, RMS was used to measure a sample of peaks and valleys.

There was a significant difference noted for the roughness between the various archwire and bracket groups (*P* = 0.044). However, no statistical significance was evident within individual groups.

The ICE-FRPC group had the highest roughness value among all the groups tested. ICE, being a monocrystalline ceramic bracket, is manufactured by milling and is very hard [23]. The milling process produces sharp corners; this, coupled with hardness, is likely to cause surface roughness on the FRPC wire.

The average roughness of the untested FRPC wire and NiTi wire was also analyzed with the AFM. There was no statistical difference between the untested FRPC and untested archwires.

For all groups tested for surface roughness, surface roughness was significantly higher in ICE-FRCP when compared to the groups with NiTi wire. This can be attributed to the higher hardness value of ICE. Further investigations is essential to validate this finding, as it is highly probable that FRPC archwires will likely be used with some form of ceramic bracket in clinical scenarios.

### The relationship between sliding friction and surface roughness

There was no correlation that could be drawn from the frictional wear and surface roughness. A higher frictional value did not translate to higher roughness values. Aforementioned observations are anticipatory and are in agreement with various studies previously carried out [25–30]. This could be attributed to many reasons; one reason could be the type of ligation. Although the type of ligation was standardized using stainless steel ligatures, it could have been more uniformed, if a steady and predictable force system could be applied via a customized jig and load cell. This arrangement would have ensured uniform seating forces of the archwire into the bracket for all the samples.

FRPC wires are a mixture of cured copolymers (Bis-EMA, TEGDMA, and glass fibers) (Table 2). Intraoral dissolution or leaching out of polymer from the composite wires might pose a potential health issue. Our study revealed exposure of fibers after frictional testing; hence, the concern would be if the wearing off of the polymer composite into the oral cavity would lead to any potential health-related issues [4, 24, 30]. Increased plaque accumulation and slow tooth movement (increased archwire-bracket friction) might be a possibility due to formation of irregular surface at fiber-exposed sites.

FRPC archwires are still in its infancy of development. Future directions in experimentation would be to include a fluid medium, like saliva, to compare wet and dry states of friction involving various orthodontic bends. Given the fact that composite wires stain easily, future focus should be on the staining characteristics of these composites wires with different beverages consumed during the course of the orthodontic treatment.

## Conclusions

Based on the discussion above, the following conclusions were drawn:

- FRPC and NiTi wires show statistically comparable frictional wear when used with ICE, Gemini, Clarity, and SmartClip brackets.
- Different archwire-bracket combinations produced different degrees of surface roughness.
- No correlation was found between frictional wear and surface roughness of the archwires of the various groups.
- FRPC wire has the potential to be used as an esthetic orthodontic archwire during the alignment stage. This is based on the comparable frictional wear and surface wear results as observed in this study.

## Authors’ original submitted files for images

Below are the links to the authors’ original submitted files for images. Authors’ original file for figure 1 Authors’ original file for figure 2 Authors’ original file for figure 3 Authors’ original file for figure 4 Authors’ original file for figure 5 Authors’ original file for figure 6 Authors’ original file for figure 7
