# Supplementary figures and images for: A new esthetic fiber-reinforced polymer composite resin archwire: a comparative atomic force microscope (AFM) and field-emission scanning electron microscope (FESEM) study
Source: Prog Orthod. 2014 May 30;15:39. doi: 10.1186/s40510-014-0039-8 (PMC4884033; doi:10.1186/s40510-014-0039-8)

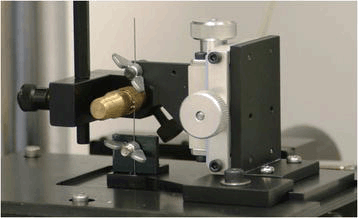

Supplement: Supplementary file 1 — Authors’ original file for figure 1 [file 40510_2014_39_MOESM1_ESM.gif]

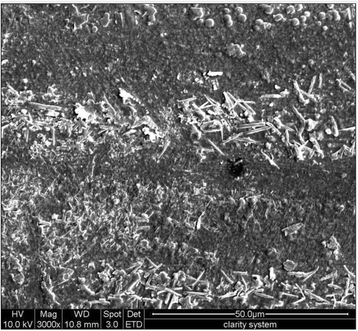

Supplement: Supplementary file 2 — Authors’ original file for figure 2 [file 40510_2014_39_MOESM2_ESM.gif]

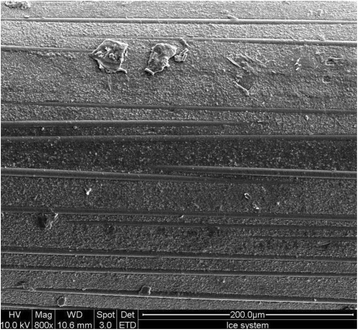

Supplement: Supplementary file 3 — Authors’ original file for figure 3 [file 40510_2014_39_MOESM3_ESM.gif]

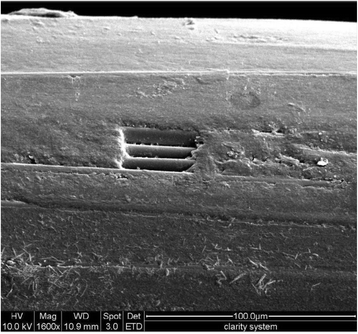

Supplement: Supplementary file 4 — Authors’ original file for figure 4 [file 40510_2014_39_MOESM4_ESM.gif]

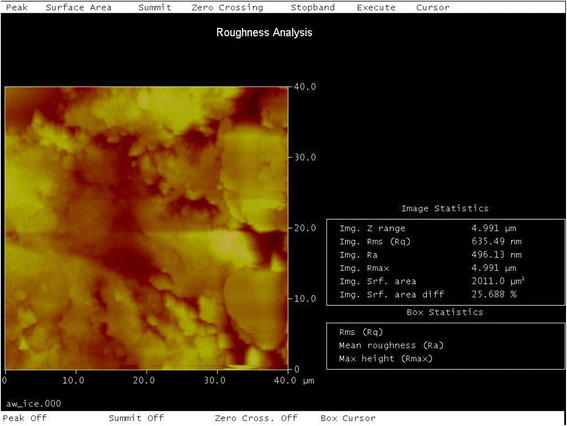

Supplement: Supplementary file 5 — Authors’ original file for figure 5 [file 40510_2014_39_MOESM5_ESM.gif]

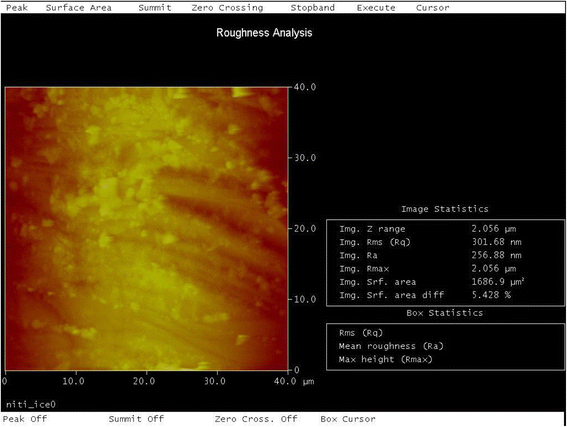

Supplement: Supplementary file 6 — Authors’ original file for figure 6 [file 40510_2014_39_MOESM6_ESM.gif]

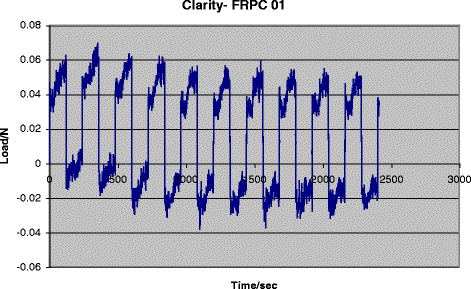

Supplement: Supplementary file 7 — Authors’ original file for figure 7 [file 40510_2014_39_MOESM7_ESM.gif]
